# Skin microbiota signature distinguishes IBD patients and reflects skin adverse events during anti-TNF therapy

**DOI:** 10.3389/fcimb.2022.1064537

**Published:** 2023-01-10

**Authors:** Zuzana Reiss, Filip Rob, Martin Kolar, Dagmar Schierova, Jakub Kreisinger, Zuzana Jackova, Radka Roubalova, Stepan Coufal, Martin Mihula, Tomas Thon, Lukas Bajer, Michaela Novakova, Martin Vasatko, Klara Kostovcikova, Natalie Galanova, Milan Lukas, Miloslav Kverka, Jana Tresnak Hercogova, Helena Tlaskalova-Hogenova, Zuzana Jiraskova Zakostelska

**Affiliations:** ^1^ Institute of Microbiology of the Czech Academy of Sciences, Prague, Czechia; ^2^ Department of Dermatovenerology, Second Faculty of Medicine, Charles University, University Hospital Bulovka, Prague, Czechia; ^3^ IBD Clinical and Research Centre ISCARE a.s., Prague, Czechia; ^4^ Department of Zoology, Faculty of Science, Charles University, Prague, Czechia; ^5^ Department of Gastroenterology and Hepatology, Institute of Clinical and Experimental Medicine, Prague, Czechia; ^6^ Institute of Medical Biochemistry and Laboratory Diagnostics, General University Hospital and First Faculty of Medicine, Charles University, Prague, Czechia; ^7^ Prof. Hercogova Dermatology, Prague, Czechia

**Keywords:** skin microbiota, IBD, 16S RNA sequencing, serum biomarker, TNF-alpha antagonist, skin adverse events

## Abstract

Crohn’s disease (CD) and ulcerative colitis (UC) are two forms of inflammatory bowel disease (IBD), where the role of gut but not skin dysbiosis is well recognized. Inhibitors of TNF have been successful in IBD treatment, but up to a quarter of patients suffer from unpredictable skin adverse events (SkAE). For this purpose, we analyzed temporal dynamics of skin microbiota and serum markers of inflammation and epithelial barrier integrity during anti-TNF therapy and SkAE manifestation in IBD patients. We observed that the skin microbiota signature of IBD patients differs markedly from healthy subjects. In particular, the skin microbiota of CD patients differs significantly from that of UC patients and healthy subjects, mainly in the retroauricular crease. In addition, we showed that anti-TNF-related SkAE are associated with specific shifts in skin microbiota profile and with a decrease in serum levels of L-FABP and I-FABP in IBD patients. For the first time, we showed that shifts in microbial composition in IBD patients are not limited to the gut and that skin microbiota and serum markers of the epithelium barrier may be suitable markers of SkAE during anti-TNF therapy.

## Introduction

The skin and mucosal surfaces are the main barriers preventing excessive contact with the external environment, and their interconnection is well established and referred to as the “gut-skin axis”. Gut microbiota alterations, impaired intestinal barrier integrity, or side effects of various drugs often manifest on the skin. The skin and its microbiota are largely neglected in many inflammatory conditions, yet they may be useful aspects to include in the search for disease risk factors and they may even serve as predictors of adverse events of anti-tumor-necrosis alpha (anti-TNF) treatment in inflammatory bowel disease (IBD).

IBD has two most prevalent forms, Crohn’s disease (CD) and ulcerative colitis (UC). IBD prevalence has been steadily increasing since 1990, reaching as high as 0.5% in Western Europe, including the Czech Republic (Jarkovský et al., 2017; Alatab et al., 2020). Despite the unknown etiology and exact pathogenesis of IBD, it is generally accepted that one of the main triggers is the aberrant immune response to commensal gut microbiota antigens in genetically susceptible individuals (Ko and Auyeung, 2014). Up to 40% of IBD patients suffer from extra-intestinal manifestations affecting various organs, including the skin (Vavricka et al., 2011). Skin manifestations of IBD can share the same histological features with manifestations in the gastrointestinal tract, e.g., granulomatous cutaneous lesions (Georgiou et al., 2006), and can arise due to immune mechanisms triggered by common antigens shared by gut bacteria and the skin (Huang et al., 2012).

TNF is a key pleiotropic cytokine involved in the pathogenesis of many inflammatory diseases, including IBD. More than two decades ago, the first anti-TNF agent was approved for the treatment of IBD and since then, TNF blockers have been widely used (Kornbluth, 1998). Interestingly, around 20% of IBD patients treated with anti-TNF develop skin adverse events (SkAE) such as eczema or psoriasiform dermatitis, referred to as paradoxical reactions (Fidder et al., 2009; Cleynen and Vermeire, 2012; Mocci et al., 2013; Nigam et al., 2021). Surprisingly, despite the widespread use of TNF blockers in IBD therapy, it is still poorly understood how the blocking of TNF influences the development of skin adverse events in IBD patients. In this case, serum biomarkers are indispensable tools which not only extent our knowledge about pathogenic mechanisms of the disease, but also reflect the spectrum of disease manifestation. Serum biomarkers are particularly important where both skin and gut are affected in the disease pathogenesis or manifestation. For example, fatty acid binding proteins, which are serum biomarkers of inflammation and epithelial barrier dysfunction (Baran et al., 2019; Lee et al., 2021), are important factors in both skin and gut involvement (Logan et al., 2022; Yin et al., 2022), and they may serve as early predictors of SkAE following anti-TNF treatment.

Many studies are mainly focused on gut microbiota analysis in IBD patients. These studies showed that levels of *Faecalibacterium prausnitzii* are decreased and the abundance of *Veillonella* and *Escherichia coli* increased in CD patients. UC patients showed decreased abundance of *Eubacterium rectale*, *Akkermansia muciniphila*, and *Roseburia* and an increase in *Escherichia coli* (Bajer et al., 2017; Pittayanon et al., 2020). Such changes in gut microbial communities can provide an opportunity for certain microbes to spread, changing thus the overall physiological setting of the microbial community. Altered behavior of microbial species and an abnormal, excessive immune response to the overgrown microbes can disrupt microbiota-mucosa interactions, allowing antigens to penetrate the mucosal barrier. This may further stimulate local and systemic immunity (Coufal et al., 2019; Caruso et al., 2020) and promote the release of mediators such as lipopolysaccharide-binding protein, matrix metalloproteinases, defensins, or fatty acid-binding proteins (Schumann and Zweigner, 1999; Winter and Wenghoefer, 2012; Clark et al., 2016; Pujada et al., 2017). A dysbiotic intestinal environment thus can be responsible for the global increase in IBD incidence and its frequent extra-intestinal manifestation on the skin (Packey and Sartor, 2008; Tlaskalová-Hogenová et al., 2011; Vavricka et al., 2011; Hold et al., 2014; Harbord et al., 2016; Sokol et al., 2017). In addition, the proinflammatory effect of IBD can also promote changes in skin microbiota composition. Specific composition of skin microbiota can then predispose for SkAE manifestation following anti-TNF treatment in IBD patients.

In this pilot study, we characterized the composition of the skin microbiota of CD and UC patients. Furthermore, we analyzed the temporal dynamics of the skin microbiota and serum biomarkers of inflammation and epithelium barrier dysfunction during anti-TNF treatment to uncover any links to the subsequent development of SkAE.

## Materials and methods

### Patients and study design

For our pilot study, 87 IBD patients were recruited at the ISCARE Clinical and Research Center for Inflammatory Bowel Disease and their skin was subsequently examined at the University Hospital Bulovka in the Czech Republic from November 2018 to December 2020. 41 healthy control subjects (HC) were recruited at the Institute of Clinical and Experimental Medicine (IKEM) from November 2018 to December 2020.

Patients were diagnosed according to the European Crohn’s and Colitis Organization (ECCO) guidelines (Maaser et al., 2019). Patients indicated for/already receiving anti-IBD biologic therapy, such as anti-TNF or anti IL-12/23 were included in the study. Only patients who were indicated for biologic treatment initiation with anti-TNF, i.e., patients who neither received any biologic treatment nor were prior exposed to anti-TNF, were recruited for the part of study monitoring the development of SkAE. In all patients, we recorded their medical history, disease duration, comorbidities, concomitant immunosuppression, response to the treatment, and various clinical and laboratory parameters at every patient’s visit (at baseline and at weeks 4, 8, 16, 22, 32, and 38 – the study endpoint) so that we could monitor the development of SkAE in anti-TNF-treated patients over time. The study endpoint refers to the end of sample collection, not to the SkAE healing. The following clinical parameters were monitored: Harvey-Bradshaw index (HBI) in patients with CD and partial Mayo score (pMayo) in patients with UC. HBI or pMayo were calculated by specialist gastroenterologists during patients’ visits based on clinical disease activity reflected by the patients’ reported outcomes and the physicians’ assessment (Harvey and Bradshaw, 1980; Schroeder et al., 1987). The following laboratory parameters were measured: C-reactive protein (CRP); fecal calprotectin (FC); ferritin; hemoglobin (Hb); platelets (PLT); white blood cells (WBC). The exclusion criteria for IBD patients were the use of antibiotics within 3 months prior sampling, prior dermatologic diseases or dermatologic extraintestinal manifestation of IBD.

Out of the 87 patients, 47 patients were not included in the study mainly because of primary non-response to anti-TNF therapy, loss of venous access, frequent nasopharyngeal infections, or due to the current COVID-19 pandemic situation. The exclusion criteria for healthy subjects were a gastrointestinal or dermatological diagnosis, concurrent or previous biologic therapy, and the use of antibiotics within 3 months before sampling. A total of 40 patients and 25 healthy controls were included in the sequencing analysis. For the analysis of skin microbiota composition in SkAE, we used 25 IBD patients on anti-TNF treatment who provided the complete sample series necessary for the longitudinal analysis, i.e., whose baseline samples were not influenced by prior exposure to any biologics. The clinical and laboratory data and baseline characteristics for all patients and controls at the time of their recruitment are summarized in **Table 1**; for a detailed overview of the characteristics of patients selected for longitudinal observation, see **Supplementary Table S1**. All study participants signed informed consent forms. This study was approved by the Ethics Committee of ISCARE (Nr2017/IIa), IKEM (G 17-06-09), and University Hospital Bulovka (21.6.2017 l8513lEK-Z).

**Table 1:** Overview of IBD patients and healthy controls parameters included in the sequencing analysis at the time of their recruitment.

	IBD patients (n = 40)		Healthy controls
	CD (n = 30)	UC (n = 10)	HC (n = 25)
Female (n)	22	7	16
Male (n)	8	3	9
Age	35 (19, 60)	34.5 (22, 63)	34.5 (20, 47)
BMI	23.41 (17.80, 35.26)	20.97 (17.30, 25.95)	22.72 (18.5, 33.51)*
Disease duration (years)	6 (1, 24)	9 (1, 20)	–
Age at diagnosis	27 (6, 55)	27 (17, 49)	–
HBI	3 (0, 10)	–	–
pMayo	–	7 (2, 12)	–

The values are shown as median with the minimum and maximum values in parentheses. Fisher exact test was used to compare the distribution of a gender nominal variables between IBD patients and HC. Mixed-effect analysis with Tukey’s multiple comparisons test was used to determine differences in Age or BMI between IBD patients and HC. n, number of participants; BMI, body mass index; HBI, Harvey-Bradshaw index; pMayo (partial Mayo score); ns, not significant;*(UC vs. HC); *p<0.05.

### Sample collection and processing

Samples were collected at each patient’s visit to capture the SkAE incidence. Skin swab samples were collected at the University Hospital Bulovka (Prague, Czech Republic). Patients were instructed not to use antiseptics for 7 days before sampling and not to bath or wash the specific skin sites for 24 hours before sampling. Samples were obtained from a 4 cm^2^ area using a sterile flocked swab (FLOQSwabsTM COPAN Diagnostics INC., USA) soaked in sterile SCF-1 buffer, as previously described (The Human Microbiome Project Consortium, 2012; Stehlikova et al., 2019a). All samples were stored at -80°C until further processing. Blood samples were collected as previously described (Coufal et al., 2019). Serum was collected at each patient’s visit and stored at −80°C until analysis. We selected serum biomarkers associated with skin and gut barrier function [e.g., E-FABP (Epidermal fatty acid-binding protein), L-FABP (Liver fatty acid-binding protein), and I-FABP (Intestinal fatty acid-binding protein)], antimicrobial response (e.g., S100A8), and inflammation (e.g., IL-18, TNF) and we quantified them using ELISA (**Supplementary Table S2**) (Coufal et al., 2019). All ELISA tests were performed according to the manufacturers’ instructions.

### Statistics

We used paired t-test to compare two experimental groups or one-way analysis of variance (ANOVA) with Dunnett’s multiple comparison test to compare multiple groups. Differences were considered statistically significant at p ≤ 0.05. Correlation analysis of selected clinical factors and biomarkers was applied and we adjusted p-values by false discovery rate. Correlation data were displayed using Spearman correlation coefficient.

### Skin microbiota sampling and processing

Sites where SkAE associated with anti-TNF treatment usually occur were sampled, namely the flexor regions – elbow and knee, retroauricular crease, skin under the lower eyelid, lumbar area, inguinal crease, and genitalia. The regions meeting the minimum sample size requirements (the threshold was set to 30 based on the amounts of particular samples we obtained), were ultimately processed for further analysis. These regions were the retroauricular crease (198 samples obtained), the lumbar area (112 samples obtained), and the inguinal crease (30 samples obtained). For a more detailed information on sample collection please see **Supplementary Figure S1**.

Extraction of total DNA from swabs was performed using the DNeasy PowerBiofilm kit (Qiagen, Germany) with minor modifications to the protocol as previously described (Stehlikova et al., 2019a; Stehlikova et al., 2019c). The subsequent PCR amplification of bacterial DNA was performed with degenerate primers 341F and 806R, which target the V3V4 region of 16S rRNA, as previously described (Stehlikova et al., 2019b). Briefly, a 25 μl reaction mixture was prepared for each sample in triplicates. PCR amplification was performed using 1X HiFi polymerase (Roche, United States), 0.4 μM primers, and 5 μl of template. Thermal cycling parameters were 33 cycles of denaturation (94°C, 3 min), annealing (55°C, 5 s), and extension (72°C, 2 min). Triplicates of PCR products were pooled to minimize random PCR bias, and the correct length of amplicons was verified by agarose gel electrophoresis. PCR amplification negative controls, extraction and sequencing positive controls (mock communities; ZymoBIOMICS Microbial Community Standard and ZymoBIOMICS Microbial Community DNA Standard, Zymo Research, USA; both in linear and logarithmic form) were processed in a similar manner.

The PCR amplicons were processed as previously described (Stehlikova et al., 2019b). Briefly, the pooled amplicons were normalized using a Sequal-Prep™ Normalization Plate (96) Kit (Illumina, United States). Adapters compatible with the MiSeq platform were ligated using the KAPA HyperPrep kit (Roche, United States), quantified, and sequenced using the MiSeq Reagent Kit v2 (2 x 300 bp) at the CEITEC Genomics Core Facility (Brno, Czech Republic). The raw sequence data are available in the Sequence Read Archive (SRA) under the accession number SUB10259106 and BioProject ID PRJNA757573.

### Skin microbiota analysis

Sequences were quality filtered with Cutadapt (version 1.15), joined with Fastq-join (1.3) and demultiplexed. The samples were rarefied at minimal sequencing depth which was 3415. The amplicon sequence variants (ASVs) were generated using DADA2, and taxonomy was assigned by vsearch against the SILVA database (release 138) with 99% similarity. Diversity analysis was performed in Qiime2 version 2020.11 (Ho et al., 2019).

Variation in alpha diversity was analyzed using Linear Mixed Models (LMM) with alpha diversity indices as the response variable (Chao1 and number of detected ASVs were log10-transformed) and patients’ identity as a random effect. Week after treatment initiation and body mass index were included as a continuous predictor, whereas sex and diagnosis were included as categorical predictors. Separate LMM was fitted for each sampling site. The significance of predictors was tested using likelihood tests and Tukey *post-hoc* comparisons. The models were fitted using the R package lme4 (Bates et al., 2015). Beta diversity was presented in principle coordinate analysis (PCoA) plots and assessed using unweighted UniFrac and Bray Curtis distances. Permutational multivariate analysis of variance (PERMANOVA) was used to confirm statistical significance between CD, UC, and healthy controls. Mixed models for longitudinal data from the R package metamicrobiomeR with individual identity as a random factor were used to examine the bacterial taxa involved in the microbiome changes between CD, UC and the HC group (Ho et al., 2019). ASVs present in < 10% of the samples were not included in these analyzes to avoid spurious results. MetamicrobiomeR package was also used for differential abundance analysis of CD^+^ vs CD^-^ and UC^+^ vs UC^-^ samples (i.e., samples of patients who either developed (+) or not developed (-) SkAE during anti-TNF treatment) (Stehlikova et al., 2019c).

## Results

### Skin microbiota diversity distinguishes CD and UC patients

To find out whether the skin microbiota differs between CD and UC patients and healthy controls (HC), we analyzed the bacterial composition of the skin at the retroauricular crease, the lumbar region, and the inguinal crease in 23 CD, 10 UC, and 21 HC using amplicon 16S rRNA gene sequencing. Our study cohort comprise 23 CD and 10 UC patients and 21 healthy controls.

**Figure 1:**
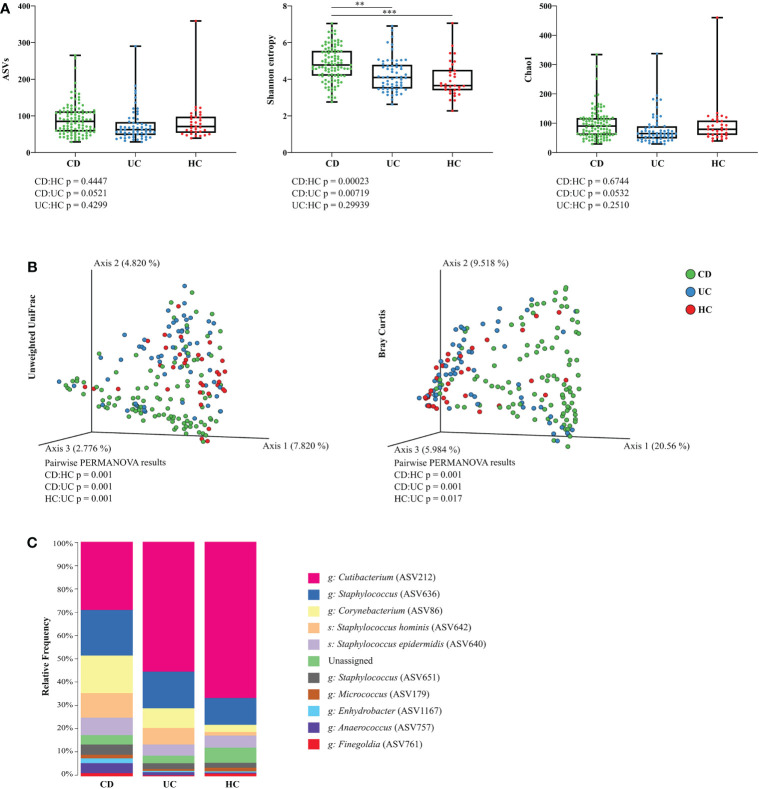
Skin microbiota diversity differs between CD, UC, and HC at the retroauricular crease. **(A)** Comparison of alpha diversity indices between CD, UC, and HC. Each point represents one sample. The differences were analyzed by Linear mixed effect models (LMM) and subsequent Tukey *post hoc* test. **(B)** Principal coordinate analysis of the Bray-Curtis distance between CD, UC, and HC. Each point represents one sample. Groups were compared by PERMANOVA. **(C)** Relative abundance of the top 10 most abundant taxa of skin microbiota at the genus and species level. In all panels: **p<0.01; ***p<0.001. ASVs, amplicon sequence variants; CD, Crohn’s disease; UC, ulcerative colitis; HC, healthy controls.

Separate models for each sampling site and diagnosis revealed that microbiota at the retroauricular crease of CD patients has significantly increased Shannon index (richness and evenness) compared to UC or HC, while the difference between UC and HC was not pronounced. The number of amplicon sequence variants (ASVs) and diversity estimator Chao1 were only slightly increased at the retroauricular crease in CD patients compared to UC patients and HC **(** **Figure 1A** **)**. Beta diversity of skin microbial communities at the reatroauricular crease, expressed as Unweighted UniFrac and Bray-Curtis distance between CD, UC, and HC showed that samples cluster by diagnosis. As a result, UC and HC were shown to be more similar to each other **(** **Figure 1B** **)**. We confirmed that these differences were not influenced by biologic treatment, sex, timescale of sampling, BMI, or the number of weeks after treatment initiation (data not shown).

Bacterial microbiota of the skin at retroauricular crease was dominated by the phyla Actinobacteria and Firmicutes, and, to a lesser extent, Proteobacteria. The most abundant bacterial genera in all samples were *Cutibacterium*, *Staphylococcus*, and *Corynebacterium*, followed by the species *Staphylococcus hominis* and *Staphylococcus epidermidis*. The relative abundance of each taxon differed between CD, UC, and HC, with CD having a relatively more diverse composition than UC or HC **(** **Figure 1C** **)**.

We observed slightly higher alpha diversity in CD compared to UC patients, and significantly higher diversity in CD over HC at the lumbar region, but not at the inguinal crease (**Supplementary Figures S2A, B**, respectively). We again confirmed that these differences were neither influenced by biologic treatment, sex, timescale of sampling, BMI, nor the number of weeks after treatment initiation (data not shown). Beta diversity of skin microbial communities at the lumbar region showed a significantly distinct microbiota profile of IBD patients when compared to healthy individuals. At the inguinal crease, microbial beta diversity of CD patients did not differ from HC but differed from that of UC patients. Beta diversity of UC patients at the inguinal crease was significantly different from that of HC (**Supplementary Figure S2C**).

Out of the three skin sites we sampled, the retroauricular crease showed the most pronounced differences in skin microbiota composition between CD, UC, and HC. For that reason, we analyzed this particular sampling site in more detail.

### CD and UC have their own microbial pattern while retaining the correlation of the shared taxa

**Figure 2:**
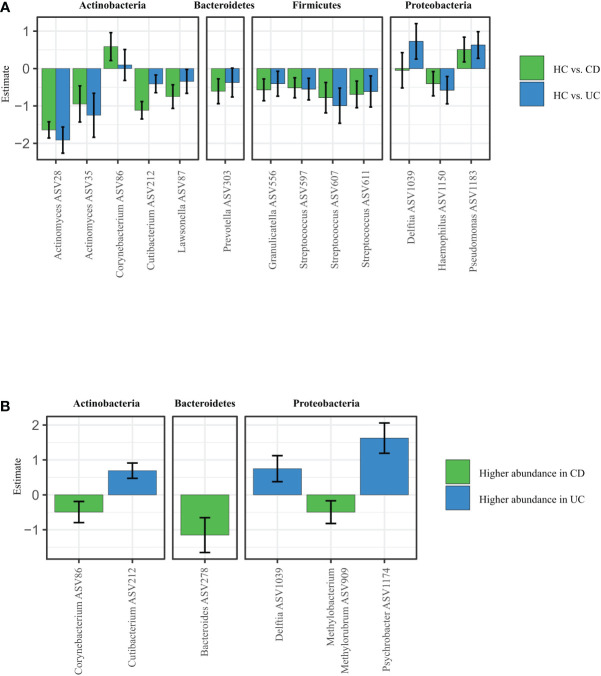
Specific microbiota pattern at the retroauricular crease distinguishes CD, UC and healthy controls. **(A)** Differential abundance analysis (DAA) of ASVs between CD and UC versus HC. Model estimates and 95% confidence intervals are shown. Positive and negative values indicate an increase and decrease in abundance, respectively, in the IBD groups compared with HC. Green color indicates ASVs abundance changes in CD compared to HC and blue color abundance changes between UC and HC. **(B)** Significant differences in taxa between CD and UC assessed by Differential abundance analysis. The taxa with significantly higher abundances in CD over UC patients are shown as negative estimates (color-coded in green), and the taxa with significantly higher abundances in UC over CD patients are shown as positive estimates (color-coded in blue). ASVs with relative abundance of more than 0.01% in all samples and detected in at least 10% samples were used in DAA analysis. CD, Crohn’s disease; UC, ulcerative colitis; HC, healthy controls.

To explain patterns distinguishing CD, UC, and HC that we observed by looking at alpha and beta diversity of our samples, we performed Differential abundance analysis (DAA). We revealed that CD patients had significantly higher abundance of *Corynebacterium* (ASV86) and *Pseudomonas* (ASV1183), and significantly lower abundance of other species such as Actinomyces (ASV28), *Cutibacterium* (ASV212), *Lawsonella* (ASV87), *Prevotella* (ASV303) or *Streptococcus* (ASV607) over HC. UC patients showed increased abundance of *Corynebacterium* (ASV86) and *Pseudomonas* (ASV1183) over HC, otherwise they followed similar pattern as CD (**Figure 2A**).

The DAA regression coefficients for CD vs. HC and UC vs. HC comparisons were closely correlated (Spearman’s correlation: rho = 0.7560, p < 0.0001), suggesting that both IBD conditions promote similar responses in the skin microbiota. At the same time, however, additional DAA comparison between the CD and HC groups showed that each for of IBD possess specific patterns of changes in ASVs abundances. For example, *Corynebacterium* (ASV86), *Bacteroides* (ASV278), or *Methylobacterium methylorubrum* (ASV909) were significantly increased in CD over UC, while *Cutibacterium* (ASV212), *Delftia* (ASV1039) or *Psychrobacter* (ASV1174) were significantly increased in UC over CD (**Figure 2B**).

### A specific skin microbiota profile might predispose IBD patients for skin side effects manifestation following anti-TNF treatment

In this section focused on monitoring SkAE following anti-TNF treatment, we used longitudinally collected samples from 25 IBD patients (17 CD and 8 UC). In this study cohort, SkAE at several skin sites affected a total of 13 CD and 4 UC patients. Different SkAE manifestations on different body sites included drug exanthema, eczema, papulopustular exanthema, herpes, and shingles. Comparing all CD and UC samples regardless of the sampled site and longitudinal nature of their collection, we observed that skin microbiota of CD patients with SkAE tended to have different proportional composition from that of CD patients without SkAE. Specifically, CD patients with SkAE had lower frequency of *Cutibacterium* (ASV212) and *Staphylococcus* (ASV636) and a higher frequency of *Corynebacterium* (ASV86), *Micrococcus* (ASV179), *Enhydrobacter* (ASV1167) or *Anaerococcus* (ASV757) when compared to CD patients without SkAE. There were no pronounced differences between UC patients with and without SkAE (**Supplementary Figure S3A**). MetamicrobiomeR-based differential abundance analysis after multiple testing corrections revealed significant association of *Actinomyces* (ASV35), *Haemophilus* (ASV1150), *Kocuria* (ASV176), *Neisseria* (ASV1068), *Staphylococcus* (ASV648), and *Streptococcus* (ASV607) with CD^+^ cohort (**Supplementary Figure S3B**), and *Actinomyces* (ASV28) with CD- cohort (**Supplementary Figure S3C**). This analysis, however, showed no significant associations for UC cohort after multiple testing corrections.

**Figure 3:**
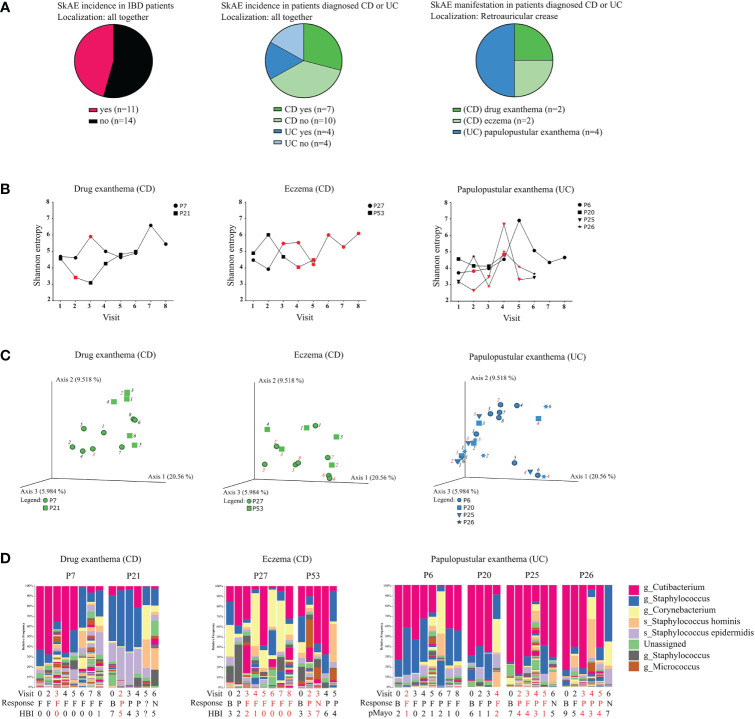
Characteristics of the IBD patients suffering from SkAE and their microbiota of retroauricular crease. **(A)** Pie charts showing patient’s distribution into groups. **(B)** Timeline of Shannon diversity changes in patients suffering from SkAE. The particular time of SkAE manifestation is color-coded in red. Graphs show a shift of Shannon entropy in particular patient’s visits. **(C)** Principal coordinate analysis of the Bray-Curtis distance between samples from patients with SkAE. All samples from one patient are represented by the same symbol, and the order of sampling is indicated by a label in italics, i.e., 1 is the first sampling. SkAE manifestation is color-coded in red. Groups were compared by PERMANOVA. **(D)** Relative abundances of the most abundant taxa of skin microbiota at the genus and species level in patients with SkAE. Visit number, response to treatment, and the disease index for CD (HBI) or UC (pMayo) are shown. P7, P21, P27, P53, P6, P20, P25, and P26 represent the patient’s code designations. CD, Crohn’s disease; UC, ulcerative colitis. Response: B, baseline; P, partial; F, full; N, no response. HBI, Harvey-Bradshaw index. pMayo, partial Mayo score.

As evaluating skin microbiota on several body sites together might be misleading, we further focused on SkAE manifestation (and therefore microbiota comparison) at the retroauricular crease, as this site showed the most pronounced differences in Shannon entropy between CD, UC, and HC. Longitudinally collected samples are particularly useful here, as they could help predict shifts in the microbial composition associated with SkAE manifestation at this skin site. We obtained samples from 2 CD patients with drug exanthema, 2 CD patients with eczema, and 4 UC patients with papulopustular exanthema at the retroauricular crease **(** **Figure 3A** **)**. We did not find any apparent skin microbiota changes that would correspond to SkAE manifestation, IBD severity, or response to the biologic treatment (i.e., loss of response during the treatment), regardless of the metric used (**Figures 3B-D**).

On the other hand, comparing the baseline of SkAE patients (CD^+^/UC^+^) with the patients who did not go on to develop SkAE (CD^-^/UC^-^) indicated a potential to predict the development of SkAE based on specific microbiota composition. Percentual taxonomic differences of ASVs between CD^+^/UC^+^ and CD^-^/UC^-^ patients and HC showed that in CD patients who later developed SkAE (CD^+^) the most abundant genus was *Staphylococcus* (ASV636) (29%), whereas in CD patients who did not develop SkAE (CD^-^), the most abundant genus was *Cutibacterium* (ASV212) (25%). Furthermore, in contrast to the CD^-^ cohort, the CD^+^ cohort was characterized by low abundance of *Staphylococcus hominis* (ASV642) (2% CD^+^; 14% CD^-^), higher abundance of *Staphylococcus epidermidis* (ASV640) (10% CD^+^; 5% CD^-^), and presence of *Dietzia* (4% in CD^+^, below 1% in CD^-^). On the other hand, CD^+^ cohort showed only negligible presence of *Anaerococcus* (ASV757) (below 1% in CD^+^, 6% in CD^-^) and *Finegoldia* (ASV761) (below 1% in CD^+^, 2% in CD^-^) compared to CD^-^ cohort. In UC patients who later developed SkAE (UC^+^), we observed higher abundance of *Anaerococcus* (ASV757) (4% in UC^+^, below 1% in UC^-^) and *Lawsonella* (ASV87) (2% in UC^+^, below 1% in UC^-^), and lower abundance of *S. hominis* (ASV642) (1% in UC^+^, 8% in UC^-^) compared to UC^-^ patients who did not develop SkAE. Interestingly, genus *Corynebacterium* (ASV86) was highly abundant in UC^-^ cohort (17%) and it was under the detection limit (set to 1%) in UC^+^ cohort. Healthy controls were shown to possess high levels of *Cutibacterium* (ASV212) (53%) and *Staphylococcus* (ASV636) (15%) and, to a lesser extent, also *Corynebacterium* (ASV86) (4%). However, *Corynebacterium* (ASV86) in HC was present to a much lesser extent than in UC^-^ patients who did not develop SkAE (4% in HC vs. 17% in UC^-^) (**Supplementary Figure S3D**). MetamicrobiomeR-based differential abundance analysis after multiple testing corrections revealed significant association of *Gemella* (ASV201), *Enhydrobacter* (ASV370), *Pseudoclavibacter* (ASV52), and *Kocuria* (ASV55) with CD^+^ cohort (**Supplementary Figure S3E**). This analysis, however, showed no significant associations for UC cohort after multiple testing corrections.

### Incidence of SkAE during anti-TNF therapy is associated with changes in the serum levels of biomarkers of epithelial barrier function

**Figure 4:**
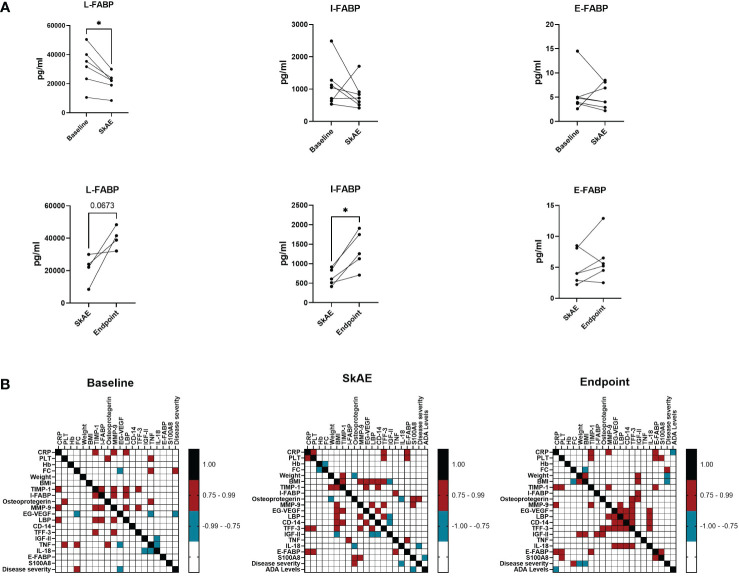
The analysis of I-FABP, L-FABP, and E-FABP in serum of patients with SkAE. **(A)** Comparison of L-FABP, I-FABP, and E-FABP levels in patients (n=7) at baseline, during the manifestation of SkAE and at the endpoint. For all panels: (^∗^ *p* < 0.05; paired t-test). **(B)** Correlation heatmap showing the Spearman’s correlation coefficient of pairwise comparison between clinical parameters and biomarkers at baseline, during SkAE incidence, and at the study endpoint (burgundy, positive correlation; turquoise, negative correlation). The heatmap was constructed in GraphPad Prism, version 8.4.3.

To gain insight into the pathogenesis of skin side effects of anti-TNF therapy, we examined different biomarkers related to skin and gut barrier function and to immune response. We analyzed 22 potential serum biomarkers at baseline, during the manifestation of SkAE, and after the healing of SkAE in serum of 7 IBD patients **(** **Supplementary Figure S4** **).** We found that a marker closely associated with epithelial integrity, L-FABP, was lowered during the manifestation of SkAE **(** **Figure 4A** **).** Conversely, I-FABP increased significantly after SkAE were healed (**Figure 4A** **)**. Moreover, we showed that there were no differences in L-FABP, I-FABP, and E-FABP at baseline between the groups of patients with and without SkAE (**Supplementary Figure S5**). Further correlation analysis of the clinical data together with potential biomarkers of SkAE identified several features associated with the occurrence of SkAE or specific to the absence of SkAE (**Figure 4B** and **Supplementary Figure S6**). Interestingly, in patients suffering SkAE we found a positive correlation of (i) I-FABP with TNF levels (r = 0.906); (ii) BMI with the levels of TIMP-1 (r = 0.786), MMP-9 (r = 0.786), LBP (r= 0.928), and EG-VGF (r = 0.808); and (iii) osteoprotegerin with IBD severity (r = 0.866), calculated as Spearman correlation coefficient. Furthermore, SkAE affected patients showed a negative correlation of (i) fecal calprotectin with hemoglobin (r = -0.821), (ii) weight with osteoprotegerin (r = 0.786), and (iii) BMI with the levels of IGFII (r = -0.786), which was not observed at baseline or at the study endpoint (**Figure 4B** **)**. In contrast, we found the same positive correlation pattern between the levels of FC and IBD severity at baseline and at the study endpoint, but not during SkAE incidence (r = 0.954 and r = 0.845) (**Figure 4B** **)**. There was no association of SkAE to blood levels of anti-TNF (data not shown).

## Discussion

The lack of tools to predict the development of skin adverse events (SkAE) after therapy with TNF blockers in IBD patients (Sridhar et al., 2018; Cossio et al., 2020) prompted us to investigate skin microbiota composition and serum biomarkers pattern in these patients.

We separately analyzed several skin localities, i.e., the retroauricular crease, the lumbar region, and the inguinal crease, to avoid inconsistency in results caused by sampling and comparing different skin ecological niches (Grice et al., 2009; Stehlikova et al., 2019a). The results of our study demonstrate that CD patients have distinct skin microbiota signature distinguishing them from UC and HC. Although differences in gut microbiota composition between CD, UC, and HC were reported (Swidsinski et al., 2008; Andoh et al., 2011; Schierova et al., 2021), the changes in skin microbiota composition we observed are rather new and unexplored. For the first time, we show that the richness and evenness represented here by Shannon entropy of skin bacterial communities at the retroauricular crease was higher in CD patients than in UC patients and HC, and that this phenomenon did not depend on the treatment (neither anti-TNF, nor anti-IL12/23), sex, or BMI. The number of observed ASVs and Chao1 index were without statistical differences in CD, UC, and HC. In addition, the lumbar region but not the inguinal crease showed significant differences between CD and HC in all three alpha diversity metrics. Although not significantly so, we observed lower Shannon entropy in CD compared to UC and HC only at the inguinal crease. This can only reflect the respective ecological niches’ characteristics (Grice et al., 2009), but further studies will be needed to confirm or refute whether these changes relate also to the particular form of the disease.

While IBD patients have generally decreased microbiota diversity in the gut (Sokol et al., 2017; Schierova et al., 2021), here we show that their skin microbiota diversity is increased compared to HC, particularly at the retroauricular crease and in the lumbar region. Importantly, the increased diversity was not due to treatment since it was observed already at baseline and it was maintained throughout the whole study. We have previously reported a similar tendency towards increased skin microbiota diversity in psoriatic skin lesions and unaffected psoriatic skin (Stehlikova et al., 2019a), while the intestinal microbiota showed the opposite trend when compared to healthy controls (Grice et al., 2009). Similarly, rosacea patients tended to have an increased abundance of various types of bacterial strains on the skin (Wang et al., 2020), while their gut microbiota richness was reduced (Chen et al., 2021). Even cirrhosis was recently shown to be associated with alterations in the skin microbiota composition (Bajaj et al., 2019).

The inguinal crease is a moist skin environment, so according to Grice et al. (2009), its diversity should exceed that of the sebaceous sites (Grice et al., 2009). This was true for UC and HC but not for CD patients, where the inguinal crease showed lower diversity than the lumbar region. From a general point of view, the skin microbiota composition of UC patients was more similar to that of HC than CD patients. Here we show that CD has a much stronger impact on the skin microbiota than UC and that each disease might affect a different microbial niche. Our finding of skin microbiota similarity between UC and HC is in line with a study focused on gut microbiota in IBD patients, where the authors observed that the difference in beta diversity between HC and UC was smaller than between HC and CD, indicating more overlapping species between HC and UC group (Sankarasubramanian et al., 2020). Despite the shared species between UC and HC, gut microbiota samples of IBD patients clustered by diagnosis and had a lower alpha diversity compared to healthy control (Bajer et al., 2017; Sokol et al., 2017; Sankarasubramanian et al., 2020; Schierova et al., 2021).

The general composition of the main bacterial phyla of IBD patients at the retroauricular crease did not markedly differ from the previously published skin microbiota of HC (Costello et al., 2009; Grice et al., 2009; Grice and Segre, 2011; Oh et al., 2014), as it was dominated by Actinobacteria and Firmicutes and to a lesser extent by Proteobacteria. The most abundant bacterial genera in all samples were *Cutibacterium* (ASV2012), *Staphylococcus* (ASV636), and *Corynebacterium* (ASV86), followed by the species *Staphylococcus hominis* (ASV642) and *Staphylococcus epidermidis* (ASV640). Cutibacteria, corynebacteria, and staphylococci are abundant colonizers of the skin, but under certain conditions can frequently cause opportunistic infections (Otto, 2010; Mayslich et al., 2021). Differential abundance analysis (DAA) of the microbiota associated with the retroauricular crease showed that compared to HC, *Corynebacterium* (ASV86) was significantly associated with CD, while *Delftia* (ASV1039) was associated with UC patients. Skin microbiota profile of both CD and UC was linked with higher abundance of *Pseudomonas* (ASV1183).

Although *Delftia* is rarely a clinically significant species, it is able to cause infections in both immunocompetent and immunocompromised individuals (Bilgin et al., 2015). Similarly, pseudomonas infections usually develop in immunocompromised individuals, but healthy people might also be sensitive to this skin commensal (Wu et al., 2011). This highlights the need to consider that the same microbe can play diverse roles in different settings. Microbial behavior can change depending on the physiological state or health of the host, for example, microbes can switch metabolite production in response to certain conditions (Otto, 2009; Ryu et al., 2014; Takahashi and Gallo, 2017). Thus, the pro-inflammatory IBD environment might impact the gut-skin axis, promoting shifts in the abundance of specific microbial species on the skin. In this way, patients might be more/less susceptible either to IBD-related skin manifestations or to skin adverse events (SkAE) often associated with anti-TNF therapy.

We hypothesized that IBD patients might have a specific skin microbiota composition that could predispose them to develop SkAE after anti-TNF treatment. These paradoxical reactions may be induced by the feedback response to the blockade of TNF, which selectively maintains type I interferon production by plasmacytoid dendritic cells (Conrad et al., 2018). By examining patients before the initiation of anti-TNF treatment, we found that certain skin microbes were associated with individuals that would later go on to develop SkAE. The relative frequency of the most abundant taxa showed that skin swab samples from CD patients who later developed SkAE (CD^+^) were predominated by *Staphylococcus* (ASV636) (29% in CD^+^ vs. 13% in CD^-^). Furthermore, the CD^+^ cohort showed a higher abundance of *Staphylococcus aureus* (ASV638) than the CD^-^ cohort (1% in CD^+^ vs. below 1% in CD^-^). The presence of *S. aureus* could be also a risk factor for atopic dermatitis development (Kong et al., 2012). Moreover, the relative abundance of staphylococci, particularly *S. aureus* and *S. epidermidis* increased in atopic dermatitis flares (Byrd et al., 2017). In agreement with Byrd et al. (2017), we observed not only an increase of *S. aureus*, but also an increase of *S. epidermidis* in CD^+^ cohort which are patients prone to develop SkAE after anti-TNF treatment (10% in CD^+^ vs. 5% in CD^-^).

The genus *Dietzia*, sometimes misidentified as *Rhodococcus* spp (Koerner et al., 2009)., is considered an opportunistic pathogen, often associated with various infections (Navaratnam et al., 2018). We observed its elevated abundance in CD^+^ over CD^-^ cohort (4% vs. below 1%, respectively), which is in concordance with another study exploring skin microbiota in atopic dermatitis (Noll et al., 2021). Similarly, we showed association of *Enhydrobacter* with skin of CD patients prone to develop SkAE and decreased abundance of *Corynebacterium* in these patients. Furthermore, *Pseudoclavibacter*, significantly associated with CD^+^ patients, was shown to be enriched in *S. aureus*-high skin psoriasis samples (Chang et al., 2018). Apart from *Enhydrobacter* and *Pseudoclavibacter*, our metamicrobiomeR analysis revealed also *Gemella* to be significantly associated with CD^+^ skin. Likewise, another study confirmed enrichment of *Gemella* in atopic dermatitis-prone individuals (Chng et al., 2016). This proposes an undeniable role of these microbes in different skin conditions. Based on our results, we suggest that skin microbes can interfere with the surrounding microbiota and may predispose IBD patients for SkAE manifestation during anti-TNF therapy.

*Anaerococcus* belongs to the group of Gram-positive anaerobic cocci (GPAC). While GPAC constitute a major part of the commensal microbiota of the skin and mucosal surfaces (Murdoch, 1998), they are frequently involved in opportunistic infections in immunocompromised or otherwise vulnerable individuals (Wall et al., 2002; Wildeboer-Veloo et al., 2007). We observed that skin microbiota in UC patients who later developed SkAE (UC^+^) showed high abundance of *Anaerococcus* species (ASV757) (4%) compared to UC^-^, where its abundance was below 1%. In contrast, *Anaerococcus* (ASV757) levels in CD^-^ patients reached 6% and they were below detection limit (below 1%) in CD^+^ patients. This suggests that microbes of one species can facilitate/protect from the manifestation of SkAE in a context-dependent manner. Although immensely interesting, these results are difficult to interpret as the analysis of predisposition to SkAE by differential abundance analysis did not provide statistically significant results due to the low number of UC+ patients, where potential differences might be masked by normal interpersonal variation. The microbiota of CD and UC patients is likely to have different settings by the very nature of the disease, and hence exerts different effects on host physiology, including, for example, the ability to respond to treatment, as well as the character of the response itself (Vasudevan et al., 2017). Although this theory is widely accepted, the power of our study is necessarily limited by the moderate number of patients with SkAE overall and the diverse forms these SkAE took.

Dysbiosis and gut-skin axis participation are common features of many inflammatory diseases like IBD or psoriasis, in the pathogenesis of which TNF plays an important role (Yost and Gudjonsson, 2009; Ruder et al., 2019). Psoriasis is a common comorbidity of IBD, with a prevalence of 3.6% in CD and 2.8% in UC (Alinaghi et al., 2020), and patients with psoriasis have a 2.5-fold higher risk of developing CD and a 1.7-fold higher risk of developing UC than the general population (Fu et al., 2018). In both IBD and psoriasis, multiple common pathogenic mechanisms have been proposed, including increased intestinal permeability (Chang et al., 2017; Stehlikova et al., 2019a) and an aberrant immune response to the microbiota (Bouma and Strober, 2003). In pediatric UC, microbial changes were observed at the sites of inflammation and mucin production was lowered even in non-inflamed parts of the bowel. A thinned mucous layer impairs the barrier function and may allow bacterial penetration, promoting the release of inflammatory markers (Alipour et al., 2016). This suggests that changes of microbiota composition and function might directly or indirectly impact the skin barrier role. In addition, Genome Wide Association Studies (GWAS) uncovered that IBD and psoriasis share 4 susceptibility loci, which contain several shared genes involved in innate and adaptive immunity (Skroza et al., 2013). The link between the two diseases has been further strengthened by the finding that they can appear to each other as paradoxical treatment-related adverse events (Lolli et al., 2015; Vlachos et al., 2016; Nehring and Przybyłkowski, 2020). As mentioned earlier, IBD patients develop multiple types of skin manifestations and patients receiving anti-TNF therapy often suffer from skin adverse events. Due to the lack of scientific evidence, however, it is not known whether TNF inhibitors affect the composition of skin microbiota. We did not observe any effect of this treatment neither on the skin nor on the gut microbiota composition (Schierova et al., 2021) and other studies report only modest differences in the gut microbiota composition after anti-TNF therapy (Bazin et al., 2018). Magnusson et al. (2016), on the other hand, showed that treatment with adalimumab improved the symptoms of UC and restored the gut microbiota of UC patients. Specifically, the authors have described an increase in the abundance of *Faecalibacterium prausnitzii* and a decrease in disease severity in responders after anti-TNF treatment (Magnusson et al., 2016). However, changes in gut microbiota after anti-TNF therapy do not have to be necessarily caused directly by anti-TNF treatment. Reversal of gut dysbiosis may simply go hand in hand with the improvement of the disease symptoms (Aldars-García et al., 2021). Together, these findings imply a dynamic interplay between the skin and gut homeostasis and highlight the important role of many gut-skin axis mediators, such as markers of inflammation or epithelial barrier function. These markers might reflect disease activity and are potentially useful for early diagnosis and prognosis.

To confirm the gut-skin axis involvement in SkAE manifestation, we searched for biomarkers related to the disruption of epithelial barrier integrity in our study cohort of IBD patients. One of the selected biomarkers, I-FABP, has been previously described to be elevated in patients with psoriasis compared to healthy controls (Stehlikova et al., 2019a; Sikora et al., 2019). However, this finding is not in line with our current results. We found a decreased production of I-FABP and L-FABP during the onset of SkAE and a subsequent increase after SkAE healing. Moreover, overexpression of E-FABP by keratinocytes has been described in patients with psoriasis (Watarai et al., 2013; Owczarczyk-Saczonek et al., 2020), but was not observed during SkAE manifestation in this study. There are two possible explanations for these unexpected results. First, the patients in this study experienced different types of SkAE, and the role of I-FABP, L-FABP, and E-FABP in the pathogenesis of drug exanthema or eczema might differ from that in psoriasis. Second, during different time points such as (i) baseline, (ii) the manifestation of SkAE, and the study endpoint (iii), different mechanism of inflammatory control might prevail. We showed a positive correlation of I-FABP with TNF levels during the occurrence of SkAE, indicating increased production of epithelial barrier disruption markers during inflammation. Osteoprotegerin (OPG) levels correlated positively with disease severity during the onset of SkAE. This finding is in agreement with previous studies that found an increased OPG in IBD patients (Moschen et al., 2005; Coufal et al., 2019). Our results highlight the hitherto unexplained relationships between the gut-skin axis serum biomarkers, anti-TNF therapy, and the development of SkAE in IBD patients. Our findings encourage further investigation of the inflammatory mechanisms in SkAE manifestation.

## Conclusions

We have shown a specific skin microbiota signature that distinguishes CD patients from UC patients and HC, suggesting that the changes in microbiota composition in IBD are not limited to the gut. This so far unique observation is another proof of the gut-skin axis interconnection in IBD, and together with other follow-up studies will pose new directions for investigation in this field. In addition, the presence or absence of certain skin microbes might predispose IBD patients for SkAE manifestation, although further investigation is necessary. The levels of serum markers of impaired epithelial barrier integrity may reflect the involvement of different inflammatory mechanisms during SkAE manifestation. Our results provide an important basis for future studies, the results of which could then serve as a prognostic tool to predict the manifestation of SkAE prior to anti-TNF treatment.

## Data availability statement

The raw sequence data are available in the BioProject repository under accession number PRJNA757573.

## Ethics statement

All study participants signed informed consent forms. This study was approved by the Ethics Committee of ISCARE (Nr2017/IIa), IKEM (G 17-06-09), and University Hospital Bulovka (21.6.2017 l8513lEK-Z). The patients/participants provided their written informed consent to participate in this study.

## Author contributions

ZJZ, HT-H, MiK, ML, FR, and JTH conceived and design the research. FR, MaK, LB, MN, MV, and ML examined the patients and healthy controls and collected samples. DS and JK performed the analysis of the sequencing data and statistics. ZR proceeded the skin swab samples and analyzed and interpreted the sequencing data. ZR and ZJZ wrote the manuscript. ZJ, RR, SC, MM, and TT analyzed the biomarkers in patient´s sera and together with ZJZ, KK, and NG interpreted the data. All authors revised and approved the final version of the manuscript.
