# Supplementary material for: Skin microbiota signature distinguishes IBD patients and reflects skin adverse events during anti-TNF therapy
Source: Front Cell Infect Microbiol. 2023 Jan 10;12:1064537. doi: 10.3389/fcimb.2022.1064537 (PMC9872723; doi:10.3389/fcimb.2022.1064537)
Supplement: Supplementary file 1 [file DataSheet_1.docx]

***Supplementary Material: METHODS***

| **CD**  **(N=24)** | | | | | **UC**  **(N=10)** | | | | **HC**  **(N=25)** | |
| --- | --- | --- | --- | --- | --- | --- | --- | --- | --- | --- |
| Female (n = 18) | | | | | Female (n = 7) | | | | Female (n = 16) | |
| Male (n = 6) | | | | | Male (n = 3) | | | | Male (n = 9) | |
|  | **CD_baseline** | **CD_endpoint** | ∆ CD p-value | CD x HC (baseline)  p value | **UC_baseline** | **UC_endpoint** | ∆ UC p-value | UC x HC (baseline)  p value | **HC_baseline** | |
| Age | 38, IQR 25 - 44  (19 – 60) | | - | - | 34.5, IQR 28.5 – 41.25  (22 – 63) | | - | - | 34.5, IQR 26.75 - 40  (20 – 47) | |
| BMI | 22.37, IQR 19.68 – 27.18  (17.8 – 30.84) | |  |  | 20.97, IQR 18.62 – 23.14  (17,3 – 25.95) | |  |  | 25.39, IQR 21.79 – 30.31  (19.34 – 33.51) | |
| Disease duration | 6, IQR 2 – 11  (1 – 24) | |  |  | 9, IQR 2.5 – 12  (1 – 20) | |  |  | - | |
| Age at diagnosis | 27, IQR 22 – 39  (6 – 55) | |  |  | 27, IQR 22 – 37.75  (17 – 49) | |  |  | - | |
| CRP | 3.75, IQR 1.7 – 6.75 (0.4 – 25.5) | 2.35, IQR 0.67 – 4.47 (0.3 – 9.2) | 0.0103 (*) | 0.0031 (**) | 2.6, IQR 0.7 – 29.9 (0.4 – 71.8) | 1.5, IQR 0.5 – 4.15 (0.3 – 14.9) | 0.3594 | 0.1279 | 1.1, IQR 0.37 – 2.72 (0.1 – 9.1) | |
| WBC | 7.9, IQR 6.87 – 9.02  (4.8 – 13.3) | 6.75, IQR 5.2 – 8.27  (3.9 – 10.4) | 0.0233  (*) | 0.0009  (***) | 11.1, IQR 5.9 – 15.45  (4.6 – 23.7) | 6.8, IQR 4.9 – 8.1  (4.4 – 9.9) | 0.0288 (*) | 0.0021 (**) | 5.65, IQR 4.75 – 6.62  (4.3 – 8.6) | |
| PLT | 346.5, IQR 260 – 415.3  (185 – 492) | 285.5, IQR 222.5 – 373  (178 – 586) | 0.0065  (**) | 0.0031  (**) | 306, IQR 261.5 – 378  (207 – 633) | 304, IQR 218.5 – 330  (179 – 430) | 0.2177 | 0.0405 (*) | 271, IQR 237.3 – 290.5  (180 – 335) | |
| Ferritin | 32.38, IQR 13.87 – 58.45  (4.16 – 191.5) | 17.01, IQR 11.96 – 44.21  (1.98 – 185) | 0.0542 | - | 23.53, IQR 13.89 – 104.1  (13.44 – 166.8) | 36.39, IQR 7.74 – 64.06)  (5.86 – 153) | 0.3594 | - | - | |
| Hb | 130.5, IQR 124.5 – 145.5  (112 – 153) | 130.5, IQR 125 – 143  (97 – 165) | 0.8043 | 0.0371 (*) | 128, IQR 108.5 – 139.5  (104 – 154) | 135, IQR 121 – 148  (110 – 156) | 0.2537 | 0.0185 (*) | 139, IQR 132.3 – 150.5  (126 – 166) | |
| FC | 300, IQR 163 – 947  (39 – 1737) | 82, IQR 40 – 287  (6 – 1974) | 0.0024  (**) | - | 1426, IQR 393.5 –3023  (51 – 5019) | 253, IQR 125.5 – 771.5  (64 – 6000) | 0.2031 | 0.0401  (*) | - | |
| HBI | 3, IQR 1 – 6.25  (0 – 8) | 3, IQR 1 – 5  (0 – 8) | 0.2634 | - | - | - | - | - | - | |
| pMayo | - | - | - | - | 7, IQR 4 – 9  (2 – 12) | 2, IQR 0.5 – 4.5  (0 – 7) | 0.0026  (**) | - | - | - |

**PATIENTS AND SAMPLE COLLECTION**

**Table S1**: Clinical and anthropometric data of CD and UC patients and healthy controls enrolled in the longitudinal cohort.

**Supplementary Table S1: Clinical and anthropometric data of CD and UC patients and healthy controls enrolled in the study.** Changes between the baseline and endpoint values of paired samples with normal distribution were evaluated by Paired t-test. Changes between the baseline values of unpaired samples with normal distribution were evaluated by Unpaired t-test; unpaired samples not having normal distribution were evaluated by Mann-Whitney test. Changes between the baseline and endpoint values of paired samples not having normal distribution were evaluated by Wilcoxon matched pairs signed-rank test. Normality of data was tested by Shapiro-Wilk normality test. Median, interquartile range (IQR), and minimal and maximal values are displayed in the table. For all comparisons: *** p < 0.001, ** p < 0.01, * p < 0.05. CD, Crohn´s disease; UC, ulcerative colitis; HC, healthy controls; BMI, body mass index; CRP, C-reactive protein; WBC, white blood cells; PLT, platelets; Hb, hemoglobin; FC, fecal calprotectin; HBI, Harvey-Bradshaw index; pMayo, partial Mayo score.

**SERUM BIOMARKERS DETECTION**

| **Biomarker** | **Abbreviation** | **Manufacturer** | **Cat. No** |
| --- | --- | --- | --- |
| Endocrine-Gland-derived Vascular Endothelial Growth Factor | EG-VEGF | R&D systems | DY1209 |
| Osteoprotegerin | OPG | R&D systems | DY805 |
| Insulin-like Growth Factor 2 | IGF2 | R&D systems | DY292 |
| Transforming Growth Factor-β1 | TGF-β1 | R&D systems | DY240 |
| Matrix Metalloproteinase 9 | MMP-9 | R&D systems | DY911 |
| Matrix Metalloproteinase 14 | MMP-14 | R&D systems | DY918 |
| Tissue Inhibitor of Metalloproteinases 1 | TIMP-1 | R&D systems | DY970 |
| Mannan-Binding Lectin | MBL | R&D systems | DY2307 |
| Soluble CD14 | CD14 | R&D systems | DY383 |
| Lipopolysaccharide-Binding Protein | LBP | R&D systems | DY870 |
| Trefoil Factor – 3 | TFF-3 | R&D systems | DY4407 |
| Tumor necrosis factor alpha | TNF-α | R&D systems | DY210 |
| Intestinal fatty acid-binding protein | I-FABP | R&D systems | DY3078 |
| Liver fatty acid-binding protein | L-FABP | HyCult Biotech | HK404 |
| Epidermal fatty acid-binding protein | E-FABP | Bio-Techne | NBP2-82538 |
| Interleukin 33 | IL-33 | R&D systems | DY3625 |
| Interleukin 18 | IL-18 | R&D systems | DY318 |
| Alpha-Defensin-1 | HD1 | R&D systems | DY8198 |
| s100a8 | s100a8 | R&D systems | DY8226 |

**Supplementary Table S2:** The list of biomarkers detected in the sera of patients and healthy controls by ELISA.


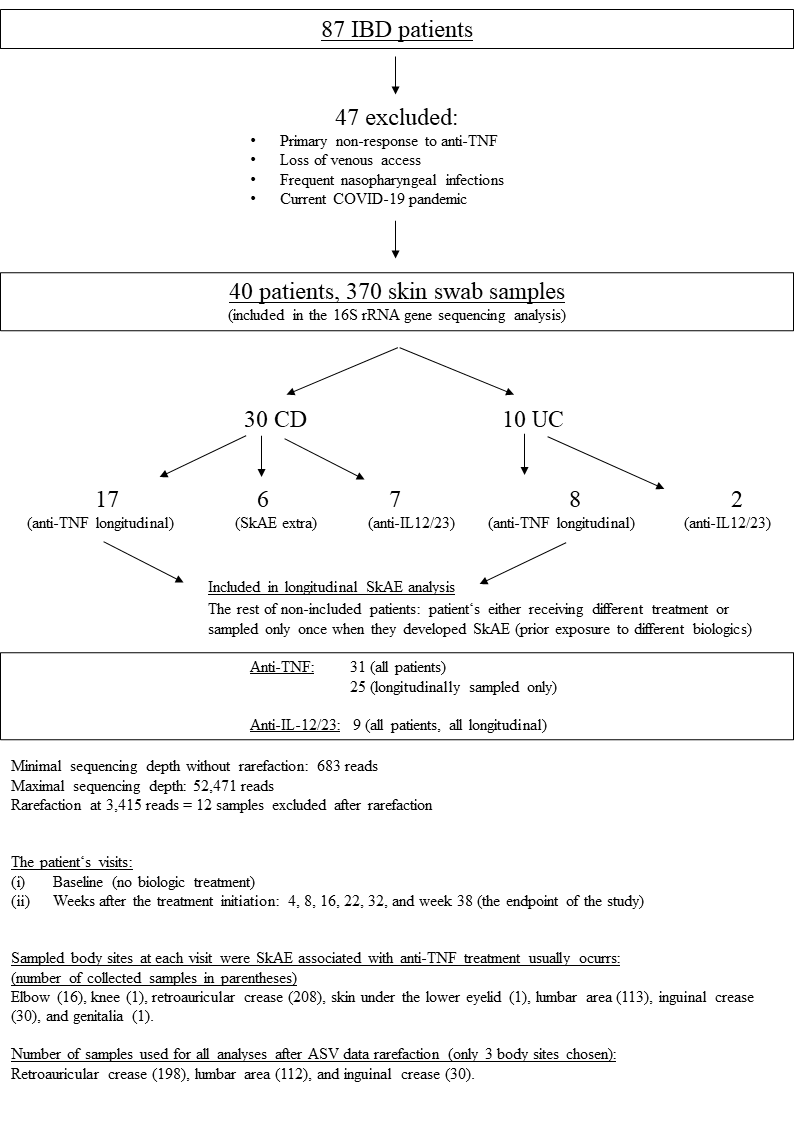


**Supplementary Figure S1:** A more detailed information on number of patients included in analyses and sample processing.

***Supplementary Material: RESULTS***


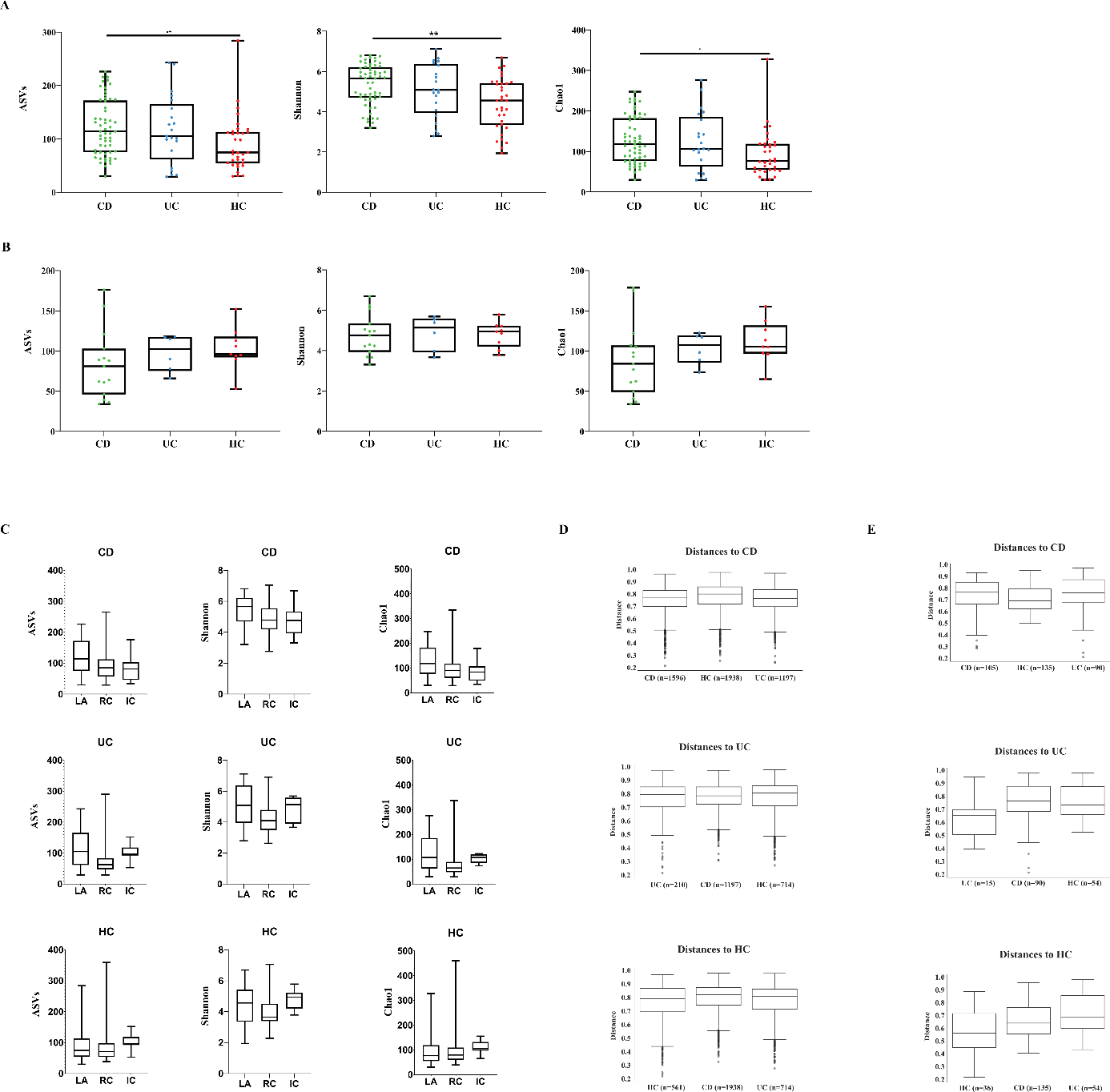


**Supplementary Figure S2: Alpha and beta diversity metrics of lumbar area and inguinal crease in CD, UC, and HC show clustering of samples according to diagnosis. (A)** Alpha diversity metrics of skin microbial communities at lumbar area. **(B)** Alpha diversity metrics of skin microbial communities at inguinal crease. **(C)** Comparison of alpha diversity indices between lumbar area (LA), retroauricular crease (RC), and inguinal crease (IC) in CD, UC, and HC. **(D)** Beta diversity metrics of skin microbial communities at lumbar area presented as Bray-Curtis distance between CD, UC, and HC. **(E)** Beta diversity metrics of skin microbial communities at inguinal crease presented as Bray-Curtis distance between CD, UC, and HC. In all panels: *p<0.05; **p<0.01; ***p<0.001.


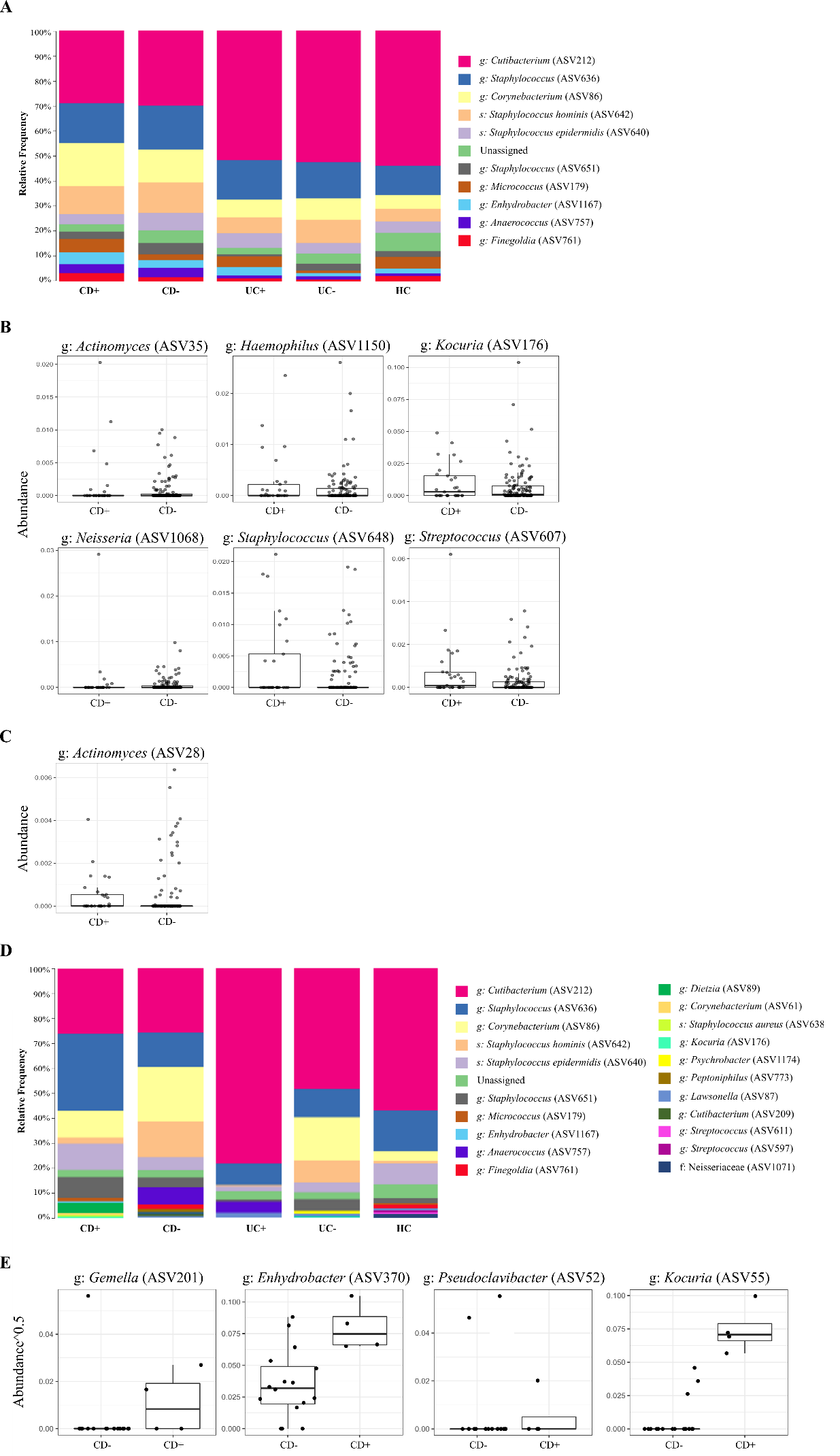


**Supplementary Figure S3: Microbiota features of SkAE cohort. (A)** Taxonomic differences between the entire cohort of CD^+^/CD^-^, UC^+^/UC^-^, and HC regardless of the sampled site and longitudinal nature of the samples collection. **(B)** MetamicrobiomeR-based differential abundance analysis of taxonomic differences between CD^+^/CD^-^ regardless of the sampled site and longitudinal nature of the samples collection. Relative abundances are square-root transformed for the purpose of graphical representation and shows ASV overrepresented in CD+ cohort. The differences between CD+/CD- were significant (p < 0.05) after multiple testing corrections. Analyzes were adjusted for systematic variation between sites and individual identity was considered as a random factor. The differences between UC^+^/UC^-^ were not significant. **(C)** MetamicrobiomeR-based differential abundance analysis of taxonomic differences between CD^+^/CD^-^ regardless of the sampled site and longitudinal nature of the samples collection. Relative abundances are square-root transformed for the purpose of graphical representation and shows ASV overrepresented in CD- cohort. The differences between CD+/CD- were significant (p < 0.05) after multiple testing corrections. Analyzes were adjusted for systematic variation between sites and individual identity was considered as a random factor. **(D)** Percentual taxonomic differences between CD^+^/CD^-^, UC^+^/UC^-^, and HC at retroauricular crease at baseline. **(E)** MetamicrobiomeR-based differential abundance analysis of taxonomic differences between CD^+^/CD^-^ at retroauricular crease at baseline. Relative abundances are square-root transformed for the purpose of graphical representation. The differences between CD^+^/CD^-^ were significant (p < 0.05) after multiple testing corrections.

**
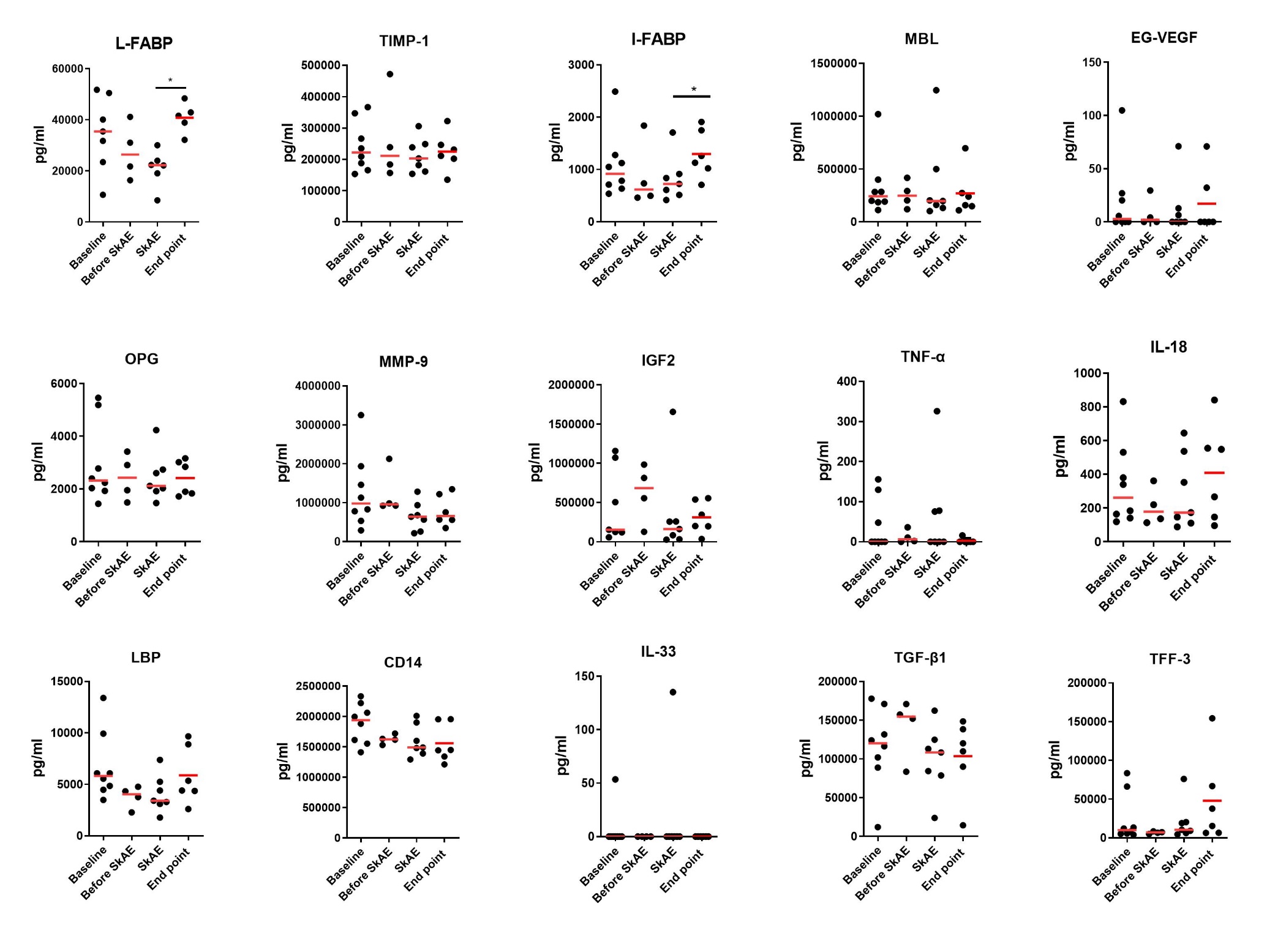
**

**Supplementary Figure S4: Analysis of serum biomarkers at baseline, just before the onset of SkAE, during the manifestation of SkAE, and at the study endpoint.** L-FABP, Liver fatty acid-binding protein; TIMP-1, Tissue Inhibitor of Metalloproteinases 1; I-FABP, Intestinal fatty acid-binding protein; MBL, Mannan-Binding Lectin; OPG, Osteoprotegerin; MMP-9 and MMP-14, Matrix Metalloproteinase 9 and 14; EG-VEGF, Endocrine-Gland-derived Vascular Endothelial Growth Factor; LBP, Lipopolysaccharide-Binding Protein; CD-14, Soluble CD14; TFF-3, Trefoil Factor – 3; TGF-b, Transforming Growth Factor-β1; TNF-a, Tumor necrosis factor alpha; IL-18 and IL-33, Interleukin 18 and 33; IGF II, Insulin-like Growth Factor 2; E-FABP, Epidermal fatty acid-binding protein; S100A8, S100 calcium-binding protein A8.


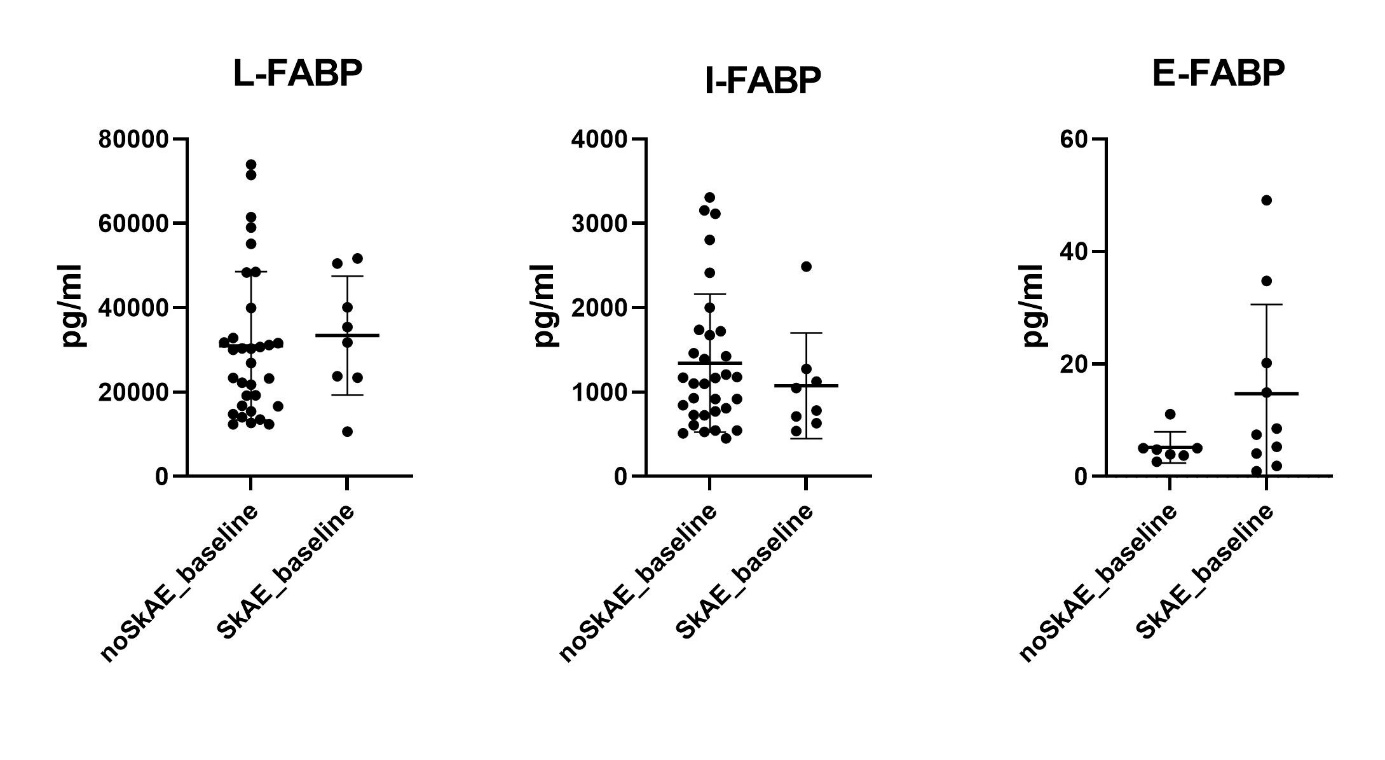


**Supplementary Figure S5: Analysis of levels of serum biomarkers between noSkAE and SkAE patients at baseline.** The levels of serum fatty acid binding proteins I-FABP, L-FABP, and E-FABP were compared between the groups of patients who showed no manifestation of SkAE during therapy (noSkAE: CD^-^, UC^-^) (n=35), and the group of patients who developed SkAE after TNFα treatment (SkAE: CD^+^, UC^+^) (n=8-10).


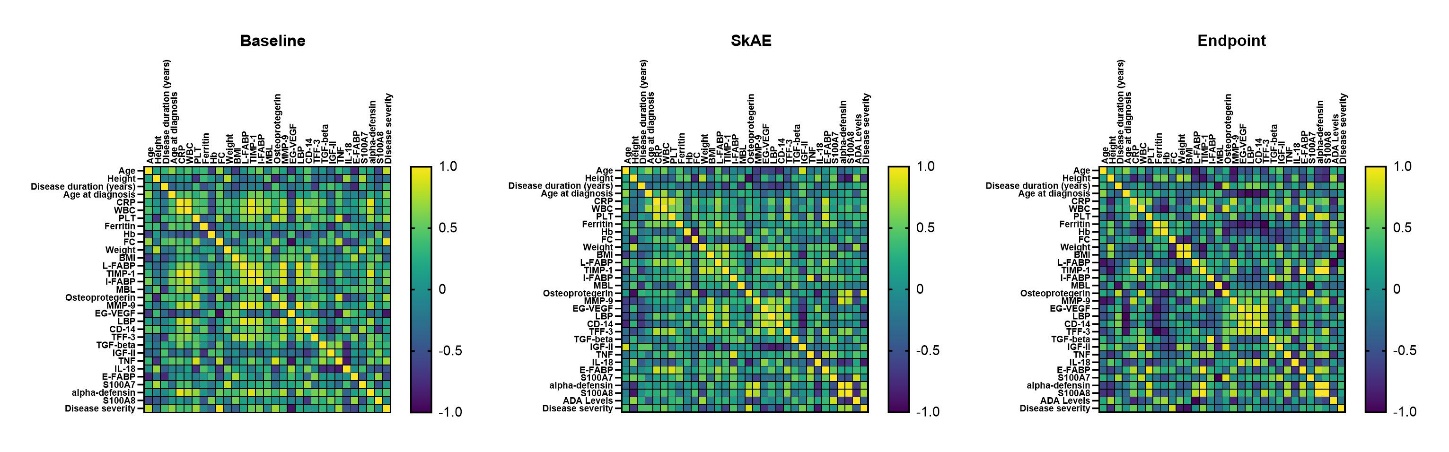


**Supplementary Figure S6**: **Heatmap analysis of clinical parameters and potential biomarkers at baseline, SkAE incidence, and at the study endpoint.** The heatmap shows Spearman’s correlation coefficient of pairwise comparison between clinical parameters and biomarkers. The heatmap was constructed in GraphPad Prism, version 8.4.3. The correlation pattern is color-coded in yellow (positive correlation) and dark blue (negative correlation).
